# Thermal imaging: A potential tool for early detection of tail lesions in pigs?

**DOI:** 10.1017/awf.2026.10079

**Published:** 2026-04-01

**Authors:** Anna Valros, Mari Heinonen, Miia Kariluoto, Frida Samuelsson, Jose Ceron, Elina Välimäki, Camilla Munsterhjelm

**Affiliations:** 1 Research Centre for Animal Welfare, Faculty of Veterinary Medicine, Department of Production Animal Medicine, University of Helsinki, Finland; 2 Thermidas Oy, Finland; 3 Department of Animal Medicine and Surgery, Veterinary School, Interdisciplinary Laboratory of Clinical Analysis of the University of Murcia (Interlab-UMU), Spain; 4 HKFoods Finland Oy, Finland

**Keywords:** Animal welfare, inflammation, saliva biomarkers, skin temperature, stress, tail biting, thermography

## Abstract

Tail biting in pigs is a serious problem both from an animal welfare and an economic perspective. Once the behaviour starts, it is important to identify it and intervene immediately to restrict the spread and risk of secondary problems, such as infections. In this study, we tested whether thermal camera imaging could be used as an aid for early detection of tail biting. We also assessed links between skin temperature, tail health and saliva biomarkers for stress and inflammation. Bitten tails were slightly warmer based on thermal imaging than non-lesioned tails. However, the difference was not sufficiently large or specific to enable its use as a practical tool in the early detection of tail lesions. The methodology, however, warrants further investigation. Shortened, but healed tails had a lower skin temperature than tails of other health categories. In combination with a lower saliva cortisol level in pigs with shortened tails, potentially indicative of chronic stress, this supports previous studies indicating chronic pain in shortened pig tails, and/or chronic stress as a result of being a victim of tail biting. These findings provide a further insight into the link between stress, infections and tail biting, while also illustrating potential for skin temperature changes to be used as an early indicator of health and welfare challenges in pigs.

## Introduction

Tail biting is an important problem from both a pig welfare and farm economic perspective (Valros 2023). The multifactorial nature of the underlying risk factors make prevention of the behaviour, not to mention the subsequent lesions very difficult to prevent. These factors include those related to genetics, management, housing and feeding of pigs (Valros 2023). Tail biting causes lesions to the victim’s tail which can vary from bruises and superficial bite marks all the way to severe loss of tissue, or even the entire tail (Valros 2023). As these lesions cause damage to both skin and underlying tissue, it is common for lesions to become infected, and secondary infection, as well as systemic spread of infection are also commonly reported (Boyle *et al.* 2022). Even those lesions appearing mild on the surface of the tail can be shown to actually be more severe, once underlying tissues are examined (Munsterhjelm *et al.* 2013).

The complexity of the risk factors and triggers for tail-biting outbreaks make early intervention crucial in the event of an outbreak, in addition to reducing risk factors (Valros 2023). However, early signs of tail biting can be hard to identify, especially in large groups of pigs, or when limited time exists for inspecting animals. Especially in young pigs most tail lesions are typically fairly mild, despite impacting welfare, for example via loss of the tail tip in undocked pigs (Valros *et al.* 2021; Munsterhjelm *et al.* 2025). Also, tail-biting behaviour has been seen to spread very much like an epidemic (Niemi *et al.* 2011), making it crucial to intervene even before severe lesions are seen, in order to stop further damage. Precision livestock farming (PLF) technology for predicting and detecting tail-biting outbreaks could be helpful in achieving early intervention. Currently, however, PLF use is limited in addressing tail-biting problems (Larsen *et al.* 2024). There is a need therefore for developing adequate and feasible tools for early identification of animals and pens showing signs of starting tail biting.

Both infections (local and systemic), and stress can cause an increase in body and surface temperatures (Teixiera *et al.* 2020). Thermographic imaging for measuring skin temperature is a non-invasive method, with potential for early detection of health challenges (Ludwig 2013). The methodology has been tested for use in production animals, to evaluate health status (Sørensen & Pedersen 2015; McManus *et al.* 2022) as well as states related to acute and chronic pain (Whittaker *et al.* 2023). As tail biting causes lesions, tissue damage and infections (Boyle *et al.* 2022), we suggest that an increase in temperature in the tail skin could be used as an early sign of mild tail biting. Indeed, Teixiera *et al.* (2020) suggested that using thermographic imaging could aid in finding bitten pigs on-farm.

Tail biting has been connected to stress, both as an underlying cause and as a result of being bitten (Munsterhjelm *et al.* 2013). Previously we were able to show a link between stress-related biomarkers and tail lesions (Valros *et al.* 2022). Cortisol is a classical biomarker for stress (Merlot *et al.* 2011; Bahnsen *et al.* 2021), while oxytocin has been the subject of increasing interest in recent years, mainly as a measure of positive experiences in production animals (Rault *et al.* 2017). Oxytocin, however, is also related to stressful situations, providing negative feedback due to increased HPA activation (Alley *et al.* 2019; Quintana & Guastella 2020). Recently, we reported a link between tail lesions and oxytocin (Valros *et al.* 2022).

Acute phase proteins, such as haptoglobin (Hp) is a well-known marker of inflammation, trauma, and infection (Ceron *et al.* 2022) and has also been suggested as a biomarker for stress (Salamano *et al.* 2008). Hp has repeatedly been shown to increase in tail-lesioned pigs (Petersen *et al.* 2002; Heinonen *et al.* 2010; Carroll *et al.* 2018). Adenosine deaminase (ADA) is a potential biomarker of immune system function (Ortin-Bustillo *et al.* 2023) and has been shown to increase in response to a standardised immune challenge in pigs (Sali *et al.* 2021). Procalcitonin (PCT) in saliva has shown potential for detection of sepsis in pigs (Lopez Martinez *et al.* 2022). We recently showed increased levels of PCT in pigs with tail lesions as compared to control pigs, suggesting a potential spread of bacteria from the tail lesion to the bloodstream (Valros *et al.* 2022).

The aim of this study was to test if thermal imaging could be used as a tool for early detection of tail-biting outbreaks. The hypothesis was that the skin temperature of a lesioned tail is higher than normal, due to local inflammation and tissue damage, even when the damage is too small to be readily observed visually in a group of pigs. To further understand if any observed skin temperature changes were due to a systemic or local reaction, or potentially to stress as a result of ongoing biting, pigs were sampled for saliva biomarkers of inflammation and stress and rectal temperature was measured. Skin temperature from ears were recorded as an additional source of information.

## Materials and methods

### Ethical approval

The study protocol was considered ethically acceptable by the University of Helsinki Viikki Campus Research Ethics Committee (Statement 13/2023).

### Study animals and housing

Data were collected from a commercial piglet-producing farm in South-Western Finland. The pigs were of Topigs Norsvin genetics (TN70 × Duroc). Based on previous results we chose to include pigs in the late nursery period in the study (approximately 8–9 weeks of age). We expected pigs of this age group to have a high prevalence of tail lesions, but the lesions would still be mainly mild (Valros *et al.* 2021; Munsterhjelm *et al.* 2025). This had been the case previously on the study farm hence our decision to select it in this instance. As the aim was to assess if thermal imaging could be used as an aid for early identification of tail biting, our emphasis was more on milder lesions since severe lesions are far easier to pick up visually from outside the pen.

Pigs were housed in identical rooms each containing 20 pens. Two of the pens in each room were sick pens and not included in the study. The remaining 18 pens per room each housed 12–15 pigs. Pens measured 3.96 × 1.76 m (length × width; 6.97 m^2^) and were equipped with a liftable roof over the resting area at the back wall (roof depth: 1.49 m). Approximately ⅔ of the pen floor was solid concrete, heated in the resting area, while the part towards the central corridor was covered with metal slats. A small amount of sawdust was spread over the solid area of the floor twice a day, and each pen was further equipped with a chain with a hockey puck attached at the end. Feed was delivered five times a day (at 0600, 0900, 1200, 1500 and 1800h) in a long trough (approximately 0.23 m per pig) as liquid meal feed. Each pen had two water nipples one above the other in the slatted area of the pen, providing *ad libitum* access to water.

### Data collection

The study included two parts, hereafter referred to as Parts 1 and 2. The specific aims of Part 1 were to assess whether a correlation exists firstly between tail (TailT) and ear (EarT) skin temperature, based on thermal imaging, and secondly between tail lesion status and biomarkers of stress, inflammation and infection. In addition, we assessed the extent to which the results from the thermal imaging correlated with rectal temperature (RectT). Part 2 sought to assess the practical applicability of thermal imaging as a tool for identifying tail lesions.

### Part 1

Sample size was calculated assuming a prevalence of any type of tail lesions of 20% and resulted in the inclusion of 500 pigs in the study (Bujang & Adnan 2016). In order to control for pen effect, the aim was to find pigs of all different tail statuses in each pen. Therefore, pens with no visible signs of tail biting (signs included lesions, hanging tails, biting behaviour) based on initial observation from the corridor were excluded. Data were collected from one room at a time, including all pigs from all pens, until 500 pigs had been sampled. For detailed information on Part 1, see Table 1.

**Table 1.** Detailed information on the timing, temperature conditions, and room, pen and pig numbers included in the two parts of this study (Parts 1 and 2)

	Part 1	Part 2
Dates	Two days in July 2023	One day in March 2024
Time	0915–1500h	1000–1300h
Outdoor temperature	15.1–16.8°C	–3.7°C
Indoor temperature	20°C	20°C
Number of rooms	3	1
Number of pens	37	15
Number of pens per room	5–18	15
Number of pigs	500 pigs	129

Assessment began with a trained assessor evaluating the tail of each pig in the pen in accordance with the scoring system shown in Table 2 (adapted from Wallgren *et al.* 2019 and Munsterhjelm *et al.* 2025). Tails were palpated and cleaned from dirt if necessary and tail length estimated visually. As tail lesion score 1 (Mild changes) was very difficult to assess objectively under the conditions on the farm, this score was not used, but tails which would have fallen into this category were considered as having no lesions (score 0). For identification with the thermal camera, pigs were then marked on their backs with running numbers using animal spray.

**Table 2.** Scoring system for pig tail health assessment, adapted from Wallgren *et al.* (2019) and Munsterhjelm *et al.* (2025). Tail lesion severity was dichotomised for analysis by collapsing categories 2–4 into category 1

Variable	Category	Definition
Tail length	0 Intact	The tail appears not to be shortened. The tip is flattened.
	1 Shortened	The tail is visibly shortened: the end is scarred and/or a part is missing. The only sign of shortening may be loss of the physiological flattening or the tip, which is thickened.
Tail lesion severity^1^	0 No lesion	There are no fresh or healing lesions, but there may be scar tissue
	2 Lesion < 5 mm	The skin is penetrated by a fresh or healing lesion smaller than 0.5 cm in size^2^
	3 Lesion 0.5–2 cm	The skin is penetrated by a fresh or healing lesion 0.5 to 2 cm in size^2^
	4 Lesion > 2 cm	The skin is penetrated by a fresh or healing lesion larger than^2^ 2 cm
Lesion Freshness^3^	0 No lesion	There is no evidence of skin-penetrating lesions, current or healed: fresh or healing lesions, or scar tissue
	1 Fresh blood or crust	There is blood or a red-coloured crust
	2 Acute open lesion	Tissue underlying the skin is visible. The tissue is moist and may be red, grey, or dark.
	3 Old crust	There is a dark-coloured crust
	4 Healed lesion	There is scar tissue: skin-like or light pink with a dry surface

1 The tail is categorised according to the most severe lesion present. Only fresh and healing lesions are recorded: moist or with visible secretion. If crust is present, the lesion is recorded. If the lesion has healed and only scar tissue is present, it is not recorded. As tail lesion score 1 (Mild changes) was very difficult to assess objectively under the conditions on the farm, this score was not used, but tails which would have fallen into this category were considered as having no lesions (score 0).

2 The size is the diameter of an area or the length of a linear lesion. If a part of the tail is missing, the area of the open lesion is measured.

3 Recorded only for skin-penetrating lesions, acute or healed. All Freshness categories visible are recorded irrespectively of their size.

A subset of pigs in each pen was selected for rectal temperature measurement and saliva sampling. The aim was to identify one pig with an intact tail with no lesion, and at least one pig representing each different tail lesion severity score (see Table 2) from every pen. As we were interested in early detection of tail biting, pigs with acute lesions were selected, i.e. not those showing signs of fully healed, but shortened tails. The first 2–6 pigs per pen (median = 3) filling these criteria were selected. In two pens, no lesioned pigs could be found, and only one pig with an intact tail from each was included. The subset used for measuring rectal temperature and saliva sampling included 37 pigs (19 females/16 castrates/2 of unknown sex) with no lesion, 42 (18/23/1, respectively) pigs with tail lesion score 2 and 33 pigs (17/14/2, respectively) with tail lesion score 3. As there were very few pigs with severe lesions (score 4, in total 4 pigs in the full data-set), only one such female pig was included in this subsample.

The pigs were guided as gently as possible using handling boards to limit their movement. Then their RectT was measured, and a saliva sample obtained as per the method described in Valros *et al.* (2022), modified from Lopez-Arjona *et al.* (2020). Pieces of polypropylene sponges were clipped to metal forceps and gently inserted into the pig’s mouth, unless the pig started to chew on their own initiative. The pigs were allowed to chew on the sponge for about 30 s. Once the sponge was clearly wet, it was placed in a Salivette tube (Sarstedt, Germany). Tubes were kept cool and centrifuged the day after sampling at 3,000 × g at 4°C for 10 min. Saliva samples were stored at –80°C until analysis. Samples were sent for analysis in a cold box layered with dry ice to the Interdisciplinary Laboratory of Clinical Analysis (Interlab-UMU) at the University of Murcia (Spain).

After a pen had been processed as described, pigs were given 10 min to calm down prior to the onset of thermal imaging. Imaging was performed from the corridor of the room, at an approximate distance of 1 to 1.5 m from the animals, with an assistant aiding in identifying the pigs, and if necessary, moving them to a spot in the pen where the image could be readily obtained. The assistant did not touch the pigs’ tail or ears at any point.

Thermal images of the tails and ears were taken with the Thermidas IRT-384 VET Tablet -thermal camera system (Thermidas, Finland) and the images were analysed with the Thermidas Remote VET -analysing software. The instantaneous field of view (IFOV) for this camera model is 1.89 mrad and the D:S ≈ 529:1. For an exact temperature measurement, 3 pixels is needed, which will bring the D:S to around 58:1. For a distance of 1 m, the minimum object size is 1.72 mm; if the distance is 1.5 m, the minimum object size is 2.58 mm. The thermal camera was calibrated to operate within a temperature range of –15 to 50°C, with the emissivity set to 0.98. The thermal resolution of the camera is 384 × 288 pixels, the sensitivity (Non-Uniformity Temperature Emissivity Drift [NETD]) is < 0.05°C at 30°C, the accuracy is ± 2°C and the field of view (FOV) is 41.5° × 31.1°. The ambient conditions were similar in all imaging areas, so the parameters for ambient temperature and humidity were automatically measured by the camera and were not included in the analysing process.

### Part 2

The data collection process was reversed to simulate a situation where a producer aims to identify early signs of tail biting using the thermal camera. This part was thus based on a more subjective methodology. Pens were selected as per Part 1 and detailed information regarding Part 2 is given in Table 1.

First, one pen at a time was inspected using the thermal camera in a similar manner as described for Part 1, to identify pigs with tails and ears that were visually and subjectively observed as warmer than others in the same pen, as well as control pigs. Pigs were assessed based on the thermal image as either having tail or ears of normal temperature, or of increased temperature. A subjective scale from 0–4 was used, whereby 4 meant a clearly warmer tail or ears than pen-mates (evident difference in thermal image colour as compared to pen-mates), and 1–3 slightly to moderately increased temperature as based on the thermal image. Study pigs were numbered using animal spray after which the same trained observers as in Part 1 assessed their tails (see Table 2), blinded to the thermal image scoring.

### Analysis of thermal images

**Figure 1.**
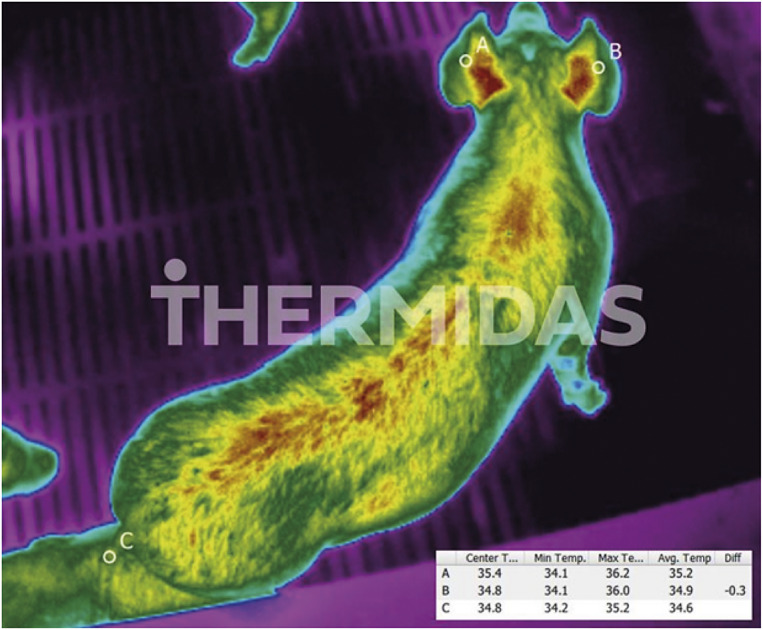
Thermal image of a pig, with regions of interest (ROIs) indicated by circles: pig’s ears (A and B) and tail (C) with temperature measurements displayed.

All images used in Part 1 were processed and analysed with the Thermidas VET 1.4.2.2 Imager analysing software. The images were analysed with the Rainbow High Contrast colour scheme to achieve optimal contrast for detecting temperature differences. The measurement areas were marked with Region of Interest (ROI) areas. The selected areas (see Figure 1) consisted of the centre of the ears and the centre length of the tail, as to minimise any imaging artefacts due to skin-on-skin contact with the areas of measurement. The ROI-areas consisted of 3 thermal pixels, from which the analysing software calculated a mean. The mean was displayed as a temperature in degrees Celsius on the image, yielding three measurement values for each animal.

### Assays for biomarkers

Oxytocin and haptoglobin were analysed using direct competitive AlphaLisa with a monoclonal antibody (López-Arjona *et al.* 2020; Contreras-Aguilar *et al.* 2021), procalcitonin was analysed with indirect competitive AlphaLisa with a monoclonal antibody (López-Martínez *et al.* 2022) and cortisol using a monoclonal antibody (López-Arjona *et al.* 2020). A Spectrophotometric automated assay (Adenosine Deaminase assay kit, Diazyme Laboratories [USA]; Tecles *et al.* 2018; Contreras-Aguilar *et al.* 2020) was used for analysing ADA.

### Data handling and statistical analyses

#### General

IBM SPSS® Statistics 29.0.0 was used for data preparation and statistical analyses. An alpha below 0.05 was considered to indicate statistical significance, and below 0.1 a trend. Normality of continuous variables was investigated using the diagnostics available in the Explore-package.

### Data processing and preliminary analysis

#### Part 1

Tail lesion severity (hereafter Tail lesion) was dichotomised for all analyses due to low prevalence of severe findings meaning all lesion categories were then collapsed into a new category 1 (‘lesioned’). The percentage of animals in the pen with a lesioned tail was calculated as a measure of social environment. EarT represented the value from the right side only, given that one left ear observation was missing, and the remaining values showed almost perfect correlation (Spearman Coefficient r_s_ = 0.98; *P* < 0.001; n = 349). TailT and EarT were considered as both raw and within-pen centred values due to signs of a strong pen effect in initial data exploration. The time of the last saliva sampling in a pen was used as an approximate time of data collection for all animals in the pen. All salivary biomarker variables were log-transformed for use as outcomes in multivariate analyses.

Two different strategies were used to quantify tail health: either the two separate variables Tail lesion and Tail length, or a new variable Tail status (see Table 3), summarising tail health in four categories based on the original scoring, as described in Table 2.

**Table 3.** Description of the variable Tail status used in the pig tail health assessment

Category	Description	Requirements
Intact	No skin-penetrating acute lesion or evidence of tail shortening. May have scratches on intact skin or scar tissue.	Lesion Type 0 and tail Shortening = ‘Normal’
Healed, shortened	Tail shortened but fully healed	Tail Shortening = ‘Shortened’ and Lesion Freshness = ‘Healed’
Small lesion	Non-healed skin-penetrating lesion up to 0.5 cm	Lesion Type 2
Large lesion	Non-healed skin-penetrating lesion > 0.5 cm	Lesion Type 3 or 4

Associations between tail health, RectT, EarT and TailT, time of sampling and salivary biomarkers were investigated by calculating r_s_ or applying the Independent-Samples Median test. The latter was followed by Bonferroni-corrected pair-wise comparisons, if significant.

#### Part 2

As the frequency of the subjective temperature scores 1 and 3 was very low, the original 5-category scale (0–4) for ear and tail temperatures was reduced to a 3-category scale by combining categories 1 and 2 (new score 1) and 3 and 4 (new score 2). Thus, the scores used for analysis were: 0 (normal); 1 (moderately elevated); and 2 (clearly elevated). Tail health was expressed in the analyses only in terms of Tail lesion and Tail shortening due to low frequency in some of the categories of Tail status. Univariate associations between EarT and TailT scores and tail health were investigated using the Chi-squared test.

### Multivariate analysis and evaluation of test performance

#### Part 1

Linear regression within the Generalized Linear Mixed Models package (GLMM) was used to investigate the associations between tail health and TailT or EarT and salivary biomarkers. Clustering was present within day (n = 2), room (n = 3) and pen (n = 37) and was accounted for through testing the contribution of each as random intercept in the models. The choice of eligible fixed predictors was based on data exploration and preliminary univariate analyses. A model was approved if normality and homoscedasticity of residuals could be determined. In cases where more than one model predicted an outcome, the one with the best fit according to the GLMM diagnostics was chosen.

GLMM results were applied as a test identifying a lesioned tail as defined by Tail lesion = 1. Correct classification of this test was obtained based on a logistic model predicting Tail lesion from TailT as well as any predictors included in the final GLMM. Performance of the test was further evaluated by Receiver Operating Characteristic curve (ROC) analysis. Youden’s index was calculated to identify the optimal cut-off. The calculations are described in detail in *Results*, as they are based on GLMM results.

#### Part 2

GLMM Multinomial regression was used to investigate effects of tail health on tail and ear temperature as described above. Clustering was accounted for by testing pen (n = 15) as random factor. Model performance was evaluated based on percentage of correct classification (PCC).

## Results

### Descriptive data

Descriptive data from Part 1 are presented in Table 4 for TailT, EarT, RectT and saliva biomarkers. Data on prevalence of lesioned and shortened tails in both Part 1 and Part 2 are shown in Table 5.

**Table 4.** Descriptive data on skin and rectal temperatures and salivary biomarkers from a total of 473 growing pigs included in Part 1 of the study

	n	Mean	SD	Median	Minimum	Maximum
Tail temperature (°C)	473	32.9	2.9	33.5	23.2	39.0
Ear temperature (right ear, °C)	350	32.5	4.1	34.0	22.4	38.5
Rectal temperature (°C)	114	40.0	0.3	400	39.2	40.9
Procalcitonin (PCT) (ng ml^–1^)	114	989	1,117	754	414	9,791
Adenosine deaminase (ADA) (IU L^–1^)	114	2,163	1,444	1,816	278	7,936
Haptoglobin (Hp) (ng mL^–1^)	114	1,504	1,047	1,291	221	6,641
Oxytocin (pg ml^–1^)	114	3,162	5,177	1,919	858	51,931
Cortisol (ng ml^–1^)	114	80.9	39.3	77.8	2.3	243.3

**Table 5.** Descriptive data on tail health in growing pigs included in Part 1 (n = 473) and 2 (n = 129) of the study

	Part 1 n = 473	Part 2 n = 129
Shortened tail	16.8%	29.5%
Lesioned tail	41.3%	50.4%
Tail status ^1^		
Intact	52.4%	45.0%
Small lesion	22.9%	14.7%
Large lesion	18.6%	35.7%
Healed, shortened	6.1%	4.7%

1 Tail status combines tail shortening, lesion and freshness (See Table 3).

Although only pens with signs of tail biting were included in Part 1, two of the 37 pens included no pigs with visibly lesioned tails. An average of 41.5% of the animals in a pen had a tail lesion (SD = 24.4). Pens including pigs with shortened tails were less prevalent, with no cases in 15 pens (40.5%). The median percentage of animals with shortened tails was 7.1% (range 0–92.3%). Of the shortened tails in Part 1, 54/83 (65%) were scored to also have a lesion, while 29/83 (35%) were already healed. Respectively for the tails of intact length, 150/411 (36%) had a lesion, while 261/411 (64%) did not. In Part 2, 32/38 (84%) of the shortened tails had lesions, while only 6/38 (16%) were healed. Of the intact length tails, 33/91 (36%) had lesions, while 58/91 (64%) did not.

In Part 2, percentages of animals in categories 0, 1 and 2 was 69.0, 23.3 and 7.8% for EarT; and 59.7, 34.1 and 6.2% for TailT, respectively (n = 129). Table 6 shows the division of tail lesion and length scorings within the different temperature categories for tails and ears.

**Table 6.** Distribution of different tail lesion and tail length scores within tail temperature (TailT) and Ear temperature (EarT) categories in growing pigs (n = 129), as based on subjective visual assessment. Cells show the number and the percentage of pigs per temperature category within each tail lesion and tail length type

	Tail lesion		Tail length	
	No tail lesion (n = 64)	Lesioned tail (n = 65)	Intact tail (n = 91)	Shortened tail (n = 38)
TailT category				
Normal (0)	45 (70%)	32 (49%)	61 (67%)	16 (42%)
Moderately elevated (1)	18 (28%)	26 (40%)	29 (32%)	15 (40%)
Clearly elevated (2)	1 (2%)	7 (11%)	1 (1%)	7 (18%)
EarT category				
Normal (0)	53 (83%)	36 (55%)	65 (71%)	24 (63%)
Clearly elevated (2)	7 (11%)	23 (35%)	19 (21%)	11 (29%)
Moderately elevated (1)	4 (6%)	6 (9%)	7 (8%)	3 (8%)

### Univariate analyses

#### Part 1

TailT and EarT temperature correlated strongly (r_s_ = 0.71; *P* < 0.001; n = 349). RectT showed no association with either of these using raw data but correlated weakly with centred TailT (r_s_ = 0.21; *P* = 0.020; n = 111).

The strongest association between measured temperatures (including both skin and rectal temperatures) and salivary biomarkers was between cortisol and EarT (r_s_ = 0.35; *P* < 0.001; n = 91), whereas weaker significant correlations existed between EarT and PCT (r_s_ = 0.25; *P* = 0.018; n = 91); TailT and cortisol (r_s_ = 0.20; *P* = 0.037; n = 111 for raw and r_s_ =0.27; *P* = 0.010; n = 114 for centred values).

Tail health was associated with a number of the collected physiological measures. A lesioned tail was related to a lower salivary PCT (*P* = 0.038; n = 113, Median test; median 700.3, range 414.0–979.7 ng ml^–1^ for a lesioned, and 798.6, 414.0–6521.3 ng ml^–1^ for a non-lesioned tail). As compared to a non-shortened tail, a shortened tail was cooler (mean [± SD] raw temperature 31.9 [± 3.0] vs 33.1 [± 2.8]°C; *P* = 0.022; n = 473). EarT and salivary PCT differed within the categories of Tail status (*P* = 0.04, and *P* = 0.02; n = 345 and n = 113, respectively; Median test). In pair-wise comparisons, EarT was lower in animals with a ‘Healed, shortened’ than an ‘Intact’ tail (29.9 [± 4.6] vs 32.8 [± 3.9]°C; *P* = 0.04). ADA correlated weakly with the percentage of animals with a tail lesion in the pen (r_s_ = –0.23; *P* = 0.013; n = 114). Time of data collection failed to correlate with any continuous variables (temperatures or salivary biomarkers; r_s_ < 0.1; *P* > 0.1 for all analyses).

#### Part 2

Lesioned tails had significantly higher TailT scores both for the tail (*P* = 0.002) and the ear (*P* = 0.017). Shortened tails had higher TailT scores than non-shortened (*P* < 0.001; n = 129, Chi-squared test; see Table 6 for raw data).

### Multivariate analyses and evaluation of test performance

#### Part 1

Centred TailT (n = 467), salivary ADA (n = 113) and cortisol (n = 110) were successfully modelled in the present data, with power 0.57 (R^2^ = 0.15), 0.13 (0.08) and 0.19 (0.12), respectively. Centred TailT was associated with Tail Lesion (*P* = 0.036), but only if controlling for Shortening with a coefficient of 0.41°C for a shortened tail (*P* = 0.28). The ‘Intact’ tail was predicted to be on average 0.27 (± 0.16)°C (estimated mean for non-shortened tail [± SE]) cooler than the pen average, and the tail with a lesion 0.13 (± 0.16)°C warmer. The 95% CI for the difference was 0.05–0.91°C. The performance of these results as a test predicting a tail lesion was based on a tail shortening-corrected centred TailT, which was calculated by adding 0.41°C to animals with a shortened tail. The ROC curve AUC of 0.557 (*P* = 0.035, 95% CI 0.504–0.610) indicated a poor classification performance. According to Youden’s Index the optimal cut-off of centred TailT is 0.49°C for a shortened and 0.21°C for a non-shortened tail, whereby an equal or higher temperature would indicate that the tail has a lesion. This cut-off yielded a sensitivity of 0.53 and a specificity of 0.41. No significant logistic model predicting Tail lesion could be built.

The four cases with the lowest cortisol concentrations had to be removed due to excessively low residual values. Cortisol was associated only with Shortening, which decreased the concentration (*P* < 0.001). Salivary ADA was associated with the percentage of animals with a tail lesion in the pen only (*P* = 0.002). The concentration decreased with an increasing percentage.

#### Part 2

For the data in Part 2, no satisfactorily performing model could be built.

## Discussion

In line with our hypothesis, we found tails with lesions to be significantly warmer than tails with no lesions within the same pen. The difference in predicted values showed an average of 0.4°C, with a relatively large confidence interval of 0.05–0.91°C, and only when controlling for shortening of the tail. The performance of these data combined as a predictor for the presence of a skin-penetrating tail lesion was evaluated as only slightly better than chance. It is important to keep in mind that due to limitations in the power of the analysis, there is a risk of Type I error, and as such these results should be considered preliminary. Considering that the accuracy of the camera is ± 2°C, this difference is relatively small but still supports the results of Teixiera *et al.* (2020), who reported an average difference of between approximately 2.5 and 4.5° between intact tails and those showing differing severity of lesions. These docked pigs were observed at the abattoir, and were probably more stressed than our pigs, which could potentially partly explain this difference. They were also older, possibly with more chronic tail wounds, and a higher risk for systemic infections.

Part 2 supported the results mentioned above, showing that tails with lesions were indeed more often scored as being warmer than intact tails. Of the pigs with tail lesions, approximately half were scored as having a normal temperature based on the thermal images, while 70% (based on TailT) and 83% (EarT) of pigs with no lesions showed a normal temperature. Thus, as for Part 1, this shows that tail lesions are indeed associated with an increase in tail (and ear) skin temperature, but that other causes can also increase the temperature, and lesions do not always result in an increased temperature.

Partly contradicting the results described above, as compared to a non-shortened tail, a shortened tail was cooler. This result, even though it should be interpreted with caution, as we could not confirm it in the multivariate analysis, is interesting, although it may have been caused by a pen effect: average tail temperatures showed considerable variation between pens, and shortening of tails was rather clustered, occurring only in 40.5% of the pens. Partly, however, the result is supported by the fact that in the multivariate analysis of Tail status, ears of pigs with ‘Healed, shortened’ tails specifically had lower skin temperature than of pigs with ‘Intact’ tails.

Of the shortened tails, slightly more than one-third (29/83; 35%) were already healed, which might help explain the above-mentioned discrepancy. A similar phenomenon was reported in tail-docked dairy cows. When measuring the superficial temperature of the tails of these adult cows, which had been tail docked prior to one year of age, the researchers found that tails of docked animals were significantly cooler than those of undocked cows (Troncoso *et al.* 2018). They suggested this to be due to sympathetic fibre sprouting in the tail stump, which can lead to vasoconstriction, and thus to lower surface temperatures. They further conclude that this finding, in turn, may confirm that docking results in chronic pain (Tronscoco *et al.* 2018). In our study, however, tail shortening affected TailT in a seemingly contradictory fashion, as shortened tails were more often allocated to a higher than normal skin temperature category than non-shortened tails in Part 2. This could be explained by the difference in proportions of fresh and healed lesions in the two parts of the study. As compared to Part 1, a larger proportion of the shortened tails also had fresh lesions in Part 2, with only 6/38 (16%) healed within the shortened tails.

As we assumed that tail lesions can lead to systemic and secondary local infections (Boyle *et al.* 2022), we hypothesised that an increased temperature, either skin or rectal, would correlate positively with biomarkers related to infection (PCT), inflammation (HP) and immune system function (ADA). However, we only found an expected positive correlation between EarT and PCT. This does not exclude the existence of further correlations, as the power of the regression analyses was slightly poor due to low effect size. Further, even though none of the pigs showed clear signs of ill-health, no thorough clinical examination of the animals took place which would have excluded any other pathological processes potentially causing variation in these measures. PCT is used as a biomarker for sepsis and is increased due to other infections as well (Jeandrot *et al.* 2008). Thus, this link might indicate that pigs with increased EarT were indeed facing some infection challenge. It must be noted, however, that this correlation was only weak. PCT has recently been linked to ill-health and tail lesions in pigs (Lopez Martinez *et al.* 2022; Valros *et al.* 2022). In the present study, however, we could not confirm our earlier reported finding (Valros *et al.* 2022) of a link between PCT and tail lesions. In our previous study, the pigs were selected to represent more severe lesions, with more than half of the tail lesions being of score 4 (over 2 cm). In the current study, we instead focused on lesions which we assumed not to be visible to the producer from the corridor and, consequently, only 1 pig (out of a total of 76 tail-lesioned pigs) included in the subset for saliva sampling had a lesion of score 4. This might also explain why we could not confirm previous results on haptoglobin being increased in tail-lesioned pigs (Heinonen *et al.* 2010; Valros *et al.* 2022). Both these previous studies included pigs with more severe and chronic lesions. Thus, overall, it may be postulated that the pigs included in this study did not have secondary sepsis that would have produced an increase in PCT and severe inflammation that would produce increases in Hp.

The negative correlation between salivary ADA and tail health is not in line with studies showing ADA to increase in situations of immune challenges (Sali *et al.* 2021), and stress (Contreras-Aguilar *et al.* 2019). In the current study, pigs in pens with a high prevalence of tail lesions instead had a decreased ADA level. ADA is related with B lymphocyte function (Contreras-Aguilar *et al.* 2020) and lack of increase and even decreased values have been found in ADA in situations of immunodepression (Llamas- Amor *et al.* 2025). Further studies should be undertaken to evaluate the immune system in pigs with tail biting and if some degree of immunodepression could be associated with this process.

Stress can markedly increase body temperature in pigs (see e.g. Warris *et al.* 2006). Accordingly, we found a positive correlation between saliva cortisol and both tail and ear temperature, while cortisol did not correlate with rectal temperature. Warris *et al.* (2006), on the other hand, reported a correlation between blood temperature and blood cortisol, sampled post-sticking and after exsanguination, as well as a tendency for a correlation between ear temperature based on thermal imaging at the same time-point and blood cortisol level in these pigs. Even though we attempted to handle the pigs as gently as possibly, and did not restrain them for more than a couple of minutes, which should be insufficient for saliva cortisol to increase significantly (Cook *et al.* 1996; Mormede *et al.* 2007), we cannot exclude the possibility that this correlation was due to an increase in both cortisol and temperature due to the stress of handling, or due to a mere increase in locomotion in the pens during the handling. The handling of pigs prior to thermal imaging in Part 1 of this study is a potential limitation also more generally. We needed to handle all pigs for tail lesion evaluation and individual marking before the thermal images could be taken, and as handling is always stressful for pigs, we cannot exclude that this could have caused changes in the skin temperature, which could mask or reduce a potential effect of tail lesions.

Although it is known that tail biting causes stress (Munsterhjelm *et al.* 2013), acute stress due to being bitten cannot fully explain the link between cortisol and tail health in this study: pigs with shortened tails had lower cortisol levels. One explanation, however, could be an increased chronic stress level in these pigs, many of whom had already been bitten prior to the onset of the present study, and had lost a part of their tail as a result. Chronic stress can result in hypocortisolism (Fries *et al.* 2005), a phenomenon suggested previously in connection to tail biting (Munsterhjelm *et al.* 2013; Valros *et al.* 2013). To test if tail-bitten pigs indeed suffer from chronic stress, studies to assess changes in the diurnal rhythm of cortisol (Munsterhjelm *et al.* 2010), or using corticotropic releasing hormone (CRH) - stimulation tests (Jarvis *et al.* 2006) could provide interesting approaches in futures studies.

Skin temperature, based on thermal imaging, only showed a weak correlation with rectal temperature and only for centred values of TailT. On the other hand, EarT correlated almost perfectly between right and left ear, and EarT and TailT correlated strongly, which supports the reliability of these measures. Previous studies have shown inconsistent results when correlating skin and body temperature: Zakari *et al.* (2021) obtained good correlations between infrared thermometer-based skin temperature in the ear and rectal temperature in pigs. Stukelj *et al.* (2022) reported varying levels of correlations between rectal temperature measures and ear skin temperature based on thermal camera images, depending on the technology used. Warriss *et al.* (2006) showed a good correlation between ear skin temperature based on thermal imaging and blood temperature after exsanguination. Ambient temperature can impact thermal image results strongly (Zakari *et al.* 2021), and could potentially also explain our result, as pen-level centred ear temperature indeed correlated, albeit weakly, with rectal temperature, and as the pen effect on the thermal imaging results was very strong. Another explanation for this is that there could have been between-pen differences in pig health status. Regrettably, we did not do thorough clinical checks of individual pigs, which could have helped us understand our results better. Indeed, it is recognised that thermographic imaging, even though having high potential for early detection of health issues, is non-specific and easily affected by environmental conditions (Ludwig 2013). The aim of this study was, however, not to assess the reliability of thermal imaging as an absolute measure of body temperature, but to investigate the usability of this method as a tool for early detection of tail lesions.

## Animal welfare implications and conclusion

We report here that even mildly bitten tails were slightly warmer than non-lesioned tails, based on thermal imaging. However, even though the difference is significant, and was partly supported by the results of the more applied Part 2 section of this study, such a difference will be difficult to utilise as a practical tool for early detection of tail lesions, especially as it appears to be relatively unspecific. The methodology, however, warrants further investigation. One interesting approach would be to use remote imaging over a longer period to study changes in skin temperature in individual pigs as a result of being tail bitten, as different types of tail biting and stages of lesions might cause variation in measures taken on one day only. This would also allow for a more careful assessment of the relationship between freshness of the lesion and tail skin temperature. The lower tail temperature in shortened, but healed tails, in combination with the lower cortisol level in pigs with shortened tails, potentially indicative of chronic stress, support previous studies indicating chronic pain in shortened pig tails, and/or chronic stress as a result of being a victim of tail biting.
